# Risk Assessment for Sudden Death in Epilepsy: The SUDEP-7 Inventory

**DOI:** 10.3389/fneur.2015.00252

**Published:** 2015-12-09

**Authors:** Jennifer L. Novak, Patrick R. Miller, Daniela Markovic, Sheba K. Meymandi, Christopher M. DeGiorgio

**Affiliations:** ^1^Department of Neurology, University of California Los Angeles School of Medicine, Sylmar, CA, USA; ^2^Department of Biomathematics, University of California Los Angeles School of Medicine, Sylmar, CA, USA; ^3^Department of Cardiology, University of California Los Angeles School of Medicine, Sylmar, CA, USA; ^4^Olive View-UCLA Medical Center, Sylmar, CA, USA

**Keywords:** SUDEP, SUDEP-7, heart rate variability, epilepsy, mortality, sudden death in epilepsy

## Abstract

**Background:**

Sudden unexpected death in epilepsy (SUDEP) is a major cause of death in those with drug-resistant epilepsy (DRE). There is a need for inventories and biomarkers associated with the risk for SUDEP.

**Objective:**

To explore the revised SUDEP Risk Inventory (SUDEP-7) in a cohort with DRE and determine the association with Heart Rate and other covariates.

**Methods:**

Twenty-five subjects with severe DRE were enrolled in a clinical trial for epilepsy. Baseline demographics, duration of epilepsy, seizure types, seizure frequency, seizure severity, AEDs, and vital signs were collected. Heart rate variability (HRV) was calculated from 1-h recordings of ECG. A SUDEP Risk Inventory (SUDEP-7) was administered, which included seven validated and weighted risk factors initially identified by Walczak et al. as factors associated with SUDEP risk.

**Results:**

The total score on the revised SUDEP-7 ranged from 1 to 7, mean = 3.4 (SD 1.8). The SUDEP Risk Inventory score was inversely correlated with RMSSD (Pearson *r* = −0.45, *p* = 0.027). The following variables were significantly associated with RMSSD: epilepsy duration (*p* = 0.02), age (*p* = 0.03), and developmental intellectual disability (*p* < 0.001). The correlation between RMSSD and SUDEP-7 tended to persist also after the adjustment for patient age (*r* = −0.40, *p* = 0.05). Two subjects died of SUDEP: their SUDEP-7 scores were above average and in the upper twenty-fifth and fiftieth percentiles, respectively (6 and 4, mean = 3.4).

**Conclusion:**

RMSSD, a measure of low frequency HRV, was significantly associated with SUDEP Risk Inventory (SUDEP-7) scores. Using a multivariate model, the covariates of developmental intellectual disability, age, and duration of epilepsy were also significantly associated with decreased HRV. The correlation between decreased HRV and a higher SUDEP-7 score remained unchanged even after the adjustment for patient age. The results suggest that older age, greater duration of epilepsy, and the presence of developmental intellectual disability may increase the risk of SUDEP through their direct influence on decreasing the vagus nerve-mediated HRV. Further validation of the SUDEP-7 inventory is indicated.

**Trial Registration:**

ClinicalTrials.gov, NCT00871377.

## Introduction

Sudden unexpected death in epilepsy (SUDEP) accounts for 16–36% of deaths in people with epilepsy and has an annual incidence of three to nine per 1,000 in the general epilepsy population (1). In drug-resistant epilepsy (DRE), the annual incidence of SUDEP is about one in 150 (2). Clinical risk factors have been prospectively identified in multiple studies. These risk factors include frequent generalized tonic–clonic seizures and long duration of epilepsy among others (2, 3).

There is a critical need for biomarkers and a screening inventory so patients at risk may be identified prior to death. This would create an opportunity to educate, monitor, and intervene to reduce the risk of SUDEP. In 2011, our group reported the first SUDEP Risk Inventory (SUDEP-7), which includes seven validated risk factors for SUDEP (4). The SUDEP-7 has been associated with two biomarkers of SUDEP risk: RMSSD and postictal generalized EEG suppression (PGES) (4, 5). RMSSD, the root mean square differences of successive R–R intervals, is a measure of vagus-mediated heart rate variability (HRV) and autonomic regulation of the heart. Low HRV, as measured by RMSSD, has been associated with atrial fibrillation, cardiovascular disease, and poor outcomes in patients with heart failure (6). In this report, we evaluate a revised SUDEP-7 Risk Inventory in a complete cohort of 25 patients with severe DRE. We correlate the revised SUDEP-7 with HRV, age, duration of epilepsy, and other key covariates and evaluate the associations of HRV with the individual risk factors of SUDEP-7 in bivariate fashion and in a multivariable model.

## Methods

Subjects were enrolled in a prospective double-blind crossover trial of a study of fish oil for epilepsy (clinicaltrials.gov # NCT00871377). Institutional Research Committee approval was secured prior to initiating the study, and informed consent was obtained from each subject or their guardian at enrollment. Inclusion criteria were as follows: ages 18–70; a history of localized, partial epilepsy; a history of generalizes tonic–clonic or tonic seizures with loss of consciousness; DRE with three or more simple partial, complex partial, or tonic–clonic seizures per month (1981 ILAE classification, partial onset seizures with or without loss of consciousness); prior exposure to at least one or more antiepileptic drugs at therapeutic doses alone or in combination; an EEG and/or an MRI consistent with a localization related epilepsy; and at least three seizures per month for at least 2 months prior to the study. Exclusion criteria were as follows: progressive medical, cardiac, or other illness; allergy to fish products or fish oil; history of coagulation disorder; history of non-epileptic seizures; consumption of fish oil 30 days or less prior to enrollment; any change in antiepileptic drugs 30 days or less prior to enrollment; warfarin treatment 30 days or less prior to enrollment; history of poor compliance with therapy; drug or alcohol abuse; uncountable seizures as a result of seizure clustering; and pregnancy.

At entry, subjects underwent a history and a physical exam, and their seizure calendars were reviewed and validated. A baseline seizure frequency was calculated from the validated seizure calendars. A National Hospital/Chalfont seizure severity scale was administered, and a score was calculated at baseline. Vital signs (heart rate, blood pressure, and weight) were determined and recorded. Subjects underwent a 1-h electrocardiogram in the resting, awake state in the sitting position, using a Philips Digitrak-plus 24 digital Holter monitoring system with a frequency range of 0.5–60 Hz and a sampling rate of 175 Hz (Philips Digitrak-Plus 24). Time-dependent measures of HRV were calculated as defined by Stein et al., and they included SDNN, SDANN, and RMSSD. SDNN is defined as the mean of the SDs for all R–R intervals; SDANN is defined as the SDs of all R–R intervals in successive 5-min epochs; and RMSSD is defined as the root mean square difference of successive R–R intervals (7).

For this study, a revised SUDEP-7 Risk Inventory was adapted from the original SUDEP-7 Risk Inventory. The SUDEP-7 Risk Inventory (Version 2.0) includes seven risk factors validated by Walczak et al. and weighted by multiplying the reported odds ratios by the natural log (log_e_) (3). The revised SUDEP Risk Inventory uses a modified scoring methodology to prevent overinflating subjects’ SUDEP score. Subjects with risk factor 1 (three or more tonic–clonic seizures in the last year) scored a 0 for risk factor 2 (one tonic–clonic seizures in the last year). Similarly, subjects with risk factor 4 (50 or more seizures per month) scored a 0 for risk factor 3 (one seizure in the last year).

All subjects were assessed with the SUDEP-7 Risk Inventory (SUDEP-7), and 24/25 completed HRV testing.

### Statistical Analysis

We evaluated the relationship between SUDEP-7 versus RMSSD using the Pearson correlation after confirming linearity. We reported the summary statistics for RMSSD including the mean (SD) and median by quartiles of the SUDEP-7 score and evaluate the association of RMSSD versus the SUDEP-7 quartile using the Spearman correlation (test of trend).

We compared the baseline RMSSD measure by level of each individual risk factor of SUDEP-7 using the Wilcoxon rank sum test or the *t*-test as appropriate. We evaluated the relationship between RMSSD versus the subset of the most important risk factors of SUDEP simultaneously using the non-parametric linear regression model with bootstrapping.

To evaluate the relationship between SUDEP-7 versus RMSSD while controlling for the covariates, such as age and sex, we used the linear regression model after confirming the normality and constant variance assumptions. Linearity was confirmed by fitting splines.

## Results

Twenty-five subjects with severe DRE were enrolled. Mean age was 33.0 years (SD 10.3), with 15 females and 10 males. Average duration of epilepsy was 21.5 years (SD 11.2). Patients in this cohort were highly drug resistant, with a mean seizure frequency of 25.1 seizures per month (SD 59.4).

Table 1 summarizes the clinical data for the cohort. Table 1 is divided into four quartiles depending on SUDEP-7 score. The total score on the SUDEP-7 ranged from 1 to 7, mean = 3.4 (SD 1.8) out of a maximum possible score of 10. Table 2 summarizes the core components of the SUDEP-7 inventory, and the number of subjects with each factor.

**Table 1:** **Summary of data for the cohort of patients, stratified by SUDEP Risk Inventory score (SUDEP-7)**.

Quartile by SUDEP-7 score	SUDEP-7 score	Age	Sex	Duration of epilepsy (years)	Seizures per month	RMSSD (ms)	Mean RMSSD by quartile
76–100%	7	45	M	45	6	14.9	17.6 ms SD 5.1
	6	21	M	16	60	22.3	
	6	18	F	17	24	N/A	
	5	46	F	31	3	21.4	
	5	28	F	28	20	11.8	
51–75%	4	56	F	44	5	15.1	25.6 ms SD 5.8
	4	44	M	33	6	32.0	
	4	32	M	31	3	23.8	
	4	40	F	34	3	20.9	
	4	42	F	30	30	26.4	
	4	32	M	14	3	30.4	
	4	30	M	22	5	33.6	
	4	34	F	29	5	23.5	
	4	30	M	26	15	24.5	
25–50%	3	28	F	20	8	60.3	35.8 ms SD 17.2
	3	19	M	5	60	26.5	
	3	37	M	15	300	21.3	
	2	32	F	10	10	23.3	
	2	22	F	12	4	47.5	
0–25%	1	28	F	7	7	20.9	32.0 ms SD 12.5
	1	24	M	10	12	31.3	
	1	37	F	7	5	31.5	
	1	26	F	21	10	31.0	
	1	22	F	17	10	55.4	
	1	53	F	13	13	21.7	
Mean	2.96	33.04		21.48	25.08	27.9	27.9
SD	1.59	10.3		11.20	59.35	11.8	11.8

*The mean RMSSD decreased with each increasing quartile of SUDEP-7, *p* = 0.032, trend test*.

**Table 2:** **The SUDEP Risk Inventory (SUDEP-7, version 2.0) with each risk factor, weighting, and scoring convention**.

SUDEP Risk Inventory (version 2.0)	Odds ratio	Weighting log_e_ ***×*** odds ratio	Number of subjects with each risk factor
1. More than three tonic– clonic seizures in last year	8.1	0 or 2	6
2. One or more tonic–clonic seizures in last year (if factor 1 present, score as 0)	2.4	0 or 1	9
3. One or more seizures of any type over the last 12 months (if factor 4 present, score as 0)	2.2, 3.8, 4.6	0 or 1	24
4. >50 seizures of any type per month over the last 12 months	11.5	0 or 2	3
5. Duration of epilepsy >30 years	13.9	0 or 3	7
6. Use of three or more AEDs	4.0	0 or 1	9
7. Developmental disability, I.Q. <70 or too impaired to test	5.0	0 or 2	3

**Figure 1:**
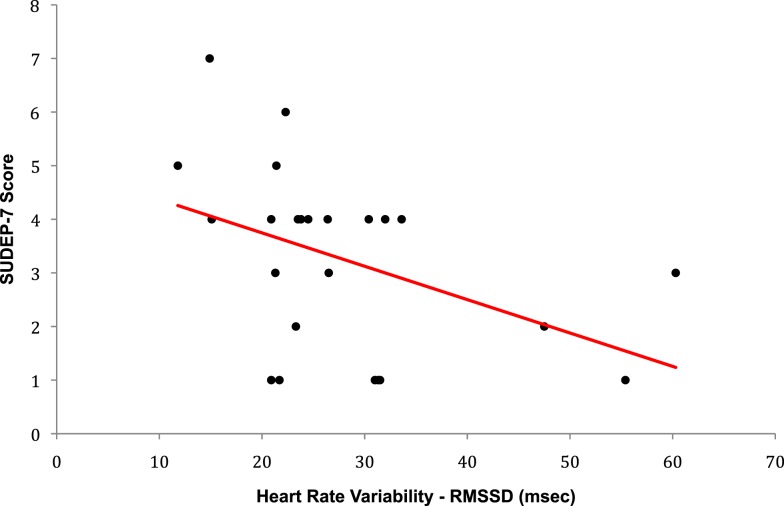
**Plot of RMSSD versus SUDEP-7 score**. The SUDEP-7 score was inversely and significantly associated with heart rate variability (RMSSD), *r* = −0.43, *p*-value = 0.0351.

**Figure 2:**
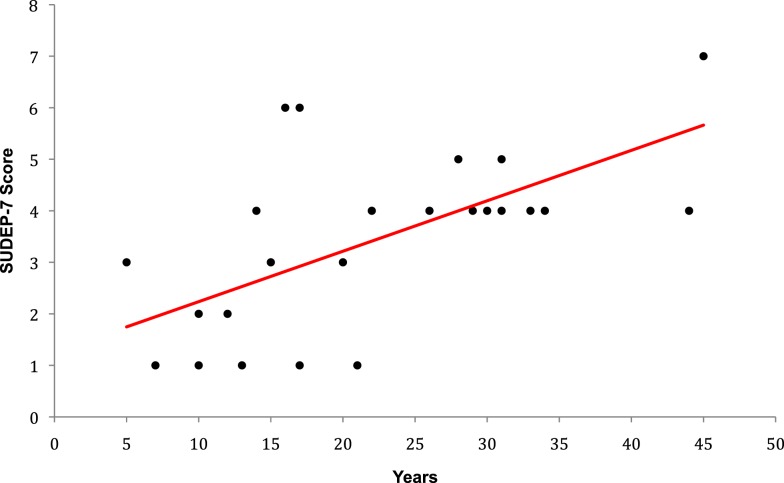
**Plot of epilepsy duration versus SUDEP-7 score**. Epilepsy duration was significantly associated with SUDEP-7 score, *r* = 0.69, *p* < 0.001.

The SUDEP-7 score was inversely correlated with RMSSD, one of the three core measures of HRV (Pearson *r* = 0.43, *p*-value = 0.035, Figure 1). Subjects in the highest SUDEP-7 quartile (scores = 5–7) had significantly lower RMSSD values than subjects in the lowest SUDEP-7 quartile (scores = 1). The mean RMSSD for the highest SUDEP-7 quartile was 17.6 ms (SD = 5.1). The mean RMSSD for the lowest SUDEP-7 quartile was 32.0 ms (SD = 12.5). RMSSD decreased significantly with each increasing quartile of SUDEP-7, *p* = 0.032, trend test, see Table 1. The SUDEP-7 score was also correlated with duration of epilepsy (*r* = 0.69, *p* < 0.001, Pearson moment correlation, Figure 2).

Subjects with developmental intellectual disabilities (mental retardation) had significantly lower baseline RMSSD values compared to subjects without developmental intellectual disabilities: mean RMSSD values for patients with developmental intellectual disabilities were 16.3 versus 29.6 ms in those without developmental intellectual disabilities (*p* = 0.026, Wilcoxon rank sum test). Age was inversely correlated with RMSSD values, with lower RMSSD values in older subjects (*r* = 0.45, *p* = 0.028). In addition, greater duration of epilepsy was correlated with decreased RMSSD (*r* = 0.65, *p* = 0.02, for persons with >20 years duration). The covariate of epilepsy duration (adjusted mean rate of change = 0.71 ms/year, *p* = 0.008, for patients with >20 years duration) remained significantly associated with decreased RMSSD even when controlled for in the same multivariable model. Older age tended to be associated with greater SUDEP-7 score, although this association did not reach statistical significance (*r* = 0.24, *p* = 0.25). The correlation between decreased RMSSD and greater SUDEP-7 score trended even after the adjustment for age (*r* = 0.40, *p* = 0.05), but any effect of age on SUDEP-7 entirely disappeared once RMSSD was accounted for in the multivariable model.

Two patients died from autopsy-confirmed SUDEP. One died during the study, and one died after exiting the study. The SUDEP-7 score for subject one was 4, and the SUDEP-7 score for subject 13 was 6. Table 3 summarizes the SUDEP-7 for the two subjects who died of SUDEP. Note, both reported three or more GTC seizures, and both were taking three or more AEDs. Subject 13 had a developmental intellectual disability (see Table 3).

**Table 3:** **SUDEP-7 scores of two subjects who died of autopsy-confirmed SUDEP**.

SUDEP-7 Risk Inventory (SUDEP-7)	Subject 1	Subject 13
1. More than three tonic–clonic seizures in last year	2	2
2. One or more tonic–clonic seizures in last year	0	0
3. One or more seizures of any type over the last 12 months	1	1
4. >50 seizures of any type per month over the last 12 months	0	0
5. Duration of epilepsy ≥30 years	0	0
6. Concurrent use of >3 antiepileptic drugs	1	1
7. Developmental intellectual disability, I.Q. <70, or too impaired to test	0	2
Total score	4	6

## Discussion

We report the revised SUDEP-7 Risk Inventory in a cohort with severe DRE. Scores in the top SUDEP-7 quartile ranged from 5 to 7. All scores in the lowest SUDEP-7 quartile were 1. Two subjects (8%) died of SUDEP. One was in the highest quartile (SUDEP-7 score = 7), and the other was in the second highest quartile (SUDEP-7 score = 4). Both subjects had above-average SUDEP-7 scores when compared with the mean score of 3.4. The high incidence of SUDEP is likely due to the high severity and longstanding duration of epilepsy in this cohort.

The revised SUDEP-7 Risk Inventory was associated with the biomarker RMSSD, a measure of vagus-mediated HRV. High SUDEP-7 scores (SUDEP-7 scores of >5) were significantly associated with low values of RMSSD. Low SUDEP-7 scores, indicating lower risk for SUDEP, were associated with higher values of vagus-mediated HRV (RMSSD). Age, duration of epilepsy, and developmental intellectual disability were all associated with reduced vagus-mediated HRV (RMSSD). The correlation of RMSSD and SUDEP-7 persisted even after the adjustment for patient age, but any effect of age on SUDEP-7 completely disappeared once RMSSD was accounted for in the multivariable model. The results suggest that older age, greater duration of epilepsy, and presence of developmental disability may increase SUDEP risk through their direct influence on vagus nerve mediated HRV (8).

This article adds to data from the initial cohort of 19 patients published in 2010 and now includes the entire study cohort of 25 subjects, with stratification of SUDEP-7 scores into quartiles, and needed modifications to the original methodology for calculating the SUDEP-7 score. This study adds to evidence that patients with developmental disability may have higher risk for SUDEP. This expanded study also provides new evidence that patients with SUDEP-7 scores in the highest quartile have significantly greater autonomic dysfunction of the heart than patients with low SUDEP-7 scores (6, 7, 9, 10). The values of RMSSD for the highest SUDEP-7 quartile averaged 17.6 ms and are similar to values in patients with heart disease at high risk for heart failure. In a recent study of 4,652 patients with cardiac risk factors who underwent HRV testing, subjects with RMSSD values similar to those in the highest SUDEP-7 quartile had significantly higher risk for heart failure. In that study, hazard ratios (HR) for heart failure ranged from a HR of 4.7:1 for RMSSD values <16 to 2.8:1 for RMSSD values of 16–26.9 ms. This indicates that patients at highest risk of SUDEP have autonomic dysfunction similar to that found in patients at high risk for heart failure.

The SUDEP-7 Risk Inventory now requires further validation in larger populations. Moseley et al. have correlated the SUDEP-7 with another biomarker, PGES. PGES was found to occur following seizures in 32.4% of 37 children monitored in a pediatric epilepsy unit (5). PGES was highly correlated with the SUDEP-7 inventory (5). Children with PGES had significantly higher SUDEP-7 scores than those without PGES SUDEP-7 score = 4.2 for those with PGES, versus 2.8 for those without PGES (5). The strong association between the SUDEP-7 inventory with a second biomarker is an important discovery and provides further independent validation.

The question of whether to include the risk factor of three or more ongoing AEDs is an ongoing question (8). Hesdorffer et al. found that the number of AEDs was not an independent predictor of SUDEP risk, after adjusting for the presence/absence of generalized tonic–clonic seizures (GTCS), age at death, and gender (11). The number of AEDs may simply reflect the severity of epilepsy and not SUDEP risk. Walczak et al. did report that three or more AEDs were an independent risk factor for SUDEP, and both subjects who died of SUDEP scored positive for the risk factor of greater than three AEDs. Further exploration regarding inclusion of three or more AEDs as a risk factor is indicated.

In summary, we report the revised SUDEP-7 Risk Inventory (SUDEP-7) in a cohort with severe DRE. The SUDEP-7 was associated with a biomarker of vagus-mediated HRV. The highest SUDEP-7 scores of 5–7 were associated with levels of autonomic function found in patients at high risk for heart failure, which reflects severely deranged autonomic control of the heart. We believe further investigation of the SUDEP-7 Risk Inventory is indicated.

## Conflict of Interest Statement

The authors declare that the research was conducted in the absence of any commercial or financial relationships that could be construed as a potential conflict of interest.
